# Human T cell lymphotropic virus type 1- associated infective dermatitis in KwaZulu Natal, South Africa

**DOI:** 10.1186/1471-5945-13-11

**Published:** 2013-10-23

**Authors:** Carol Hlela, Natalie Graham, Ahmed I Bhigjee, Graham P Taylor, Nonhlanhla P Khumalo, Anisa Mosam

**Affiliations:** 1 Division of Dermatology; Red Cross Children’s Hospital, University of Cape Town, Cape Town, South Africa; 2 Department of Dermatology; Nelson Mandela School of Medicine, University of KwaZulu Natal, Congella, Durban, South Africa; 3 Virology Laboratory, Africa Centre for Health and Population Studies, University of KwaZulu-Natal, Durban, South Africa; 4 Neurology Department, Nelson Mandela School of Medicine, University of KwaZulu Natal, Congella, Durban, South Africa; 5 Department of GU Medicine and Communicable Diseases, Imperial College, Faculty of Medicine, Norfolk Place, London, UK; 6 Division of Dermatology, Groote Schuur Hospital, Observatory, Cape Town, South Africa

## Abstract

**Background:**

The Human T cell lymphotropic virus type 1 (HTLV-1)-associated infective dermatitis (IDH), is a chronic relapsing dermatitis which usually presents in children older than 2 years. A total of 300 cases have been reported worldwide (Latin America, the Caribbean and only 5 from Senegal). Neither IDH, nor its complications have been reported from the rest of Africa. We aimed to examine the clinical and aetiological characteristics of IDH in a cohort of South African children.

**Methods:**

Attendees at the dermatology clinic at King Edward VIII Hospital, Durban underwent clinical examination. After obtaining consent those suspected of IDH had specimens taken for blood counts, immunoglobulins, serum protein electrophoresis, viral studies (including genotyping), skin swabs and stool examinations.

**Results:**

Nineteen of 60 suspected cases recruited over 3 years met the diagnostic criteria for IDH. The male-to-female ratio was 1:2; mean age 8 years (range 0.7 to 15). Dermatitis mostly affected the scalp (78.9%) and axilla (73.7%); fewer children had nasal crusting (47.4%). Mean Ig A, IgG and IgM were raised, at 3.52 g/l, 22.6 g/l and 1.38 g/l, respectively. The median CD4 cell count was 1958 cells/mm^3^. Viral genotyping of all tested samples were positive for the *Cosmopolitan, Subtype A* (HTLV-1a).

**Conclusions:**

IDH is a distinct entity which also affects South Africans. Our patients were older at presentation and the majority did not present with nasal crusting as has been described in other countries.

## Background

The human T-cell lymphotropic virus type 1 (HTLV-1)-associated infective dermatitis (IDH) was first reported in Carribean children; the incidence and pathogenesis are unknown. Somewhat reminescent of seborhoeic dermatitis, the clinical presentation of IDH is that of severe exudative dermatitis with crusting of the scalp, neck, axillae, groin, external ear, and retro-auricular areas; watery nasal discharge, and/or crusting of the anterior nares, from about 2 years of age. We identified fewer than 30 publications which report less than 300 cases of IDH worldwide; reviewed in [1] The largest series reported 50 patients from Jamaica [2]. There has only been one report of five African cases with IDH from Senegal [3]. IDH has been linked with the development of adult T cell leukemia/lymphoma (ATLL) and HTLV-1-associated myelopathy/paraparesis (HAM/TSP) [4-7]. We aimed to examine the clinical and aetiological characteristics of IDH in a cohort of South African children.

### Study subjects

Following ethical approval by the Biomedical Research Ethics Committee, University of KwaZulu Natal, we enrolled sixty individuals with suspected IDH, screened using the established criteria, Table 1[2], amongst outpatients attending dermatology clinic at King Edward VIII Hospital, Durban KwaZulu Natal (KZN). Where possible, the parents and siblings of the HTLV-1 seropositive participants were also recruited. Written informed consent was obtained from the patients for publication of this case series and the accompanying images. A detailed dermatological examination was done. Formal ophthalmologic and neurologic examinations were conducted on patients who had symptoms and/or signs of visual and neurological abnormalities respectively. Diagnosis of HAM/ TSP was made according to WHO guidelines [8].

**Table 1:** Clinical criteria for IDH diagnosis

***Major***	***Minor***
1. Eczema of scalp, axillae and groin external ear and retro-auricular areas, eyelid margins, paranasal skin and/or neck	1. Positive cultures for *Staphylococcus aureus* and/or *Beta-haemolytic streptococcus* from the skin or anterior nares
2. Chronic watery nasal discharge without other signs of rhinitis and/or crusting of the anterior nares	2. Generalized fine papular rash (in most severe cases)
3. Chronic relapsing dermatitis with prompt response to appropriate therapy but prompt recurrence on withdrawal of use of antibiotic	3. Generalized lymphadenopathy with dermatopathic lymphadenitis
4. Usual onset in early childhood	4. Anaemia
5. Human T cell lymphotropic virus type 1 antibody seropositivity	5. Elevated erythrocyte sedimentation rate
	6. Hyperimmunoglobulinaemia (IgD and IgE )
	7. Elevated CD4 count, CD8 count, and CD4/CD8 ratio

Four of the criteria required for diagnosis, with mandatory inclusion of 1,2 and 5; to meet criterion 1, at least two of the sites must be affected.

## Methods

Blood counts, measurement of immunoglobulin levels, serum protein electrophoresis, viral studies, skin swabs for bacterial culture and examination of stool samples for parasites were done. All enrolled participants (n = 60) had HTLV-1 and HIV-1 serological testing done. HIV-1 serology was performed using the Vironostika HIV-1 IMPVD Microelisa system (Biomerieux, Durham, NC) with all positives samples confirmed by a second HIV ELISA test. Serology for HTLV-1 was performed on plasma using an enzyme-immune assay (EIA) that detects anti-HTLV-1 and anti-HTLV-2 antibodies (Ab-Capture EIA Test System – Ortho-Clinical Diagnostics, Inc., Rarita, New Jersey). HTLV-1 Western blots were not available so HTLV-1 infection was confirmed by HTLV PCR. DNA was extracted from peripheral blood mononuclear cells (PBMC) of seropositive patients using the QIAamp Blood kit (Qiagen Inc., Chatsworth, CA, U.S.A). Detection of a 318-bp product or a 161-bp product confirmed the presence of HTLV-1 or HTLV-2 *pol* respectively. To sub-type HTLV-1 a fragment of LTR was amplified by nested PCR using primers 12P1/SK111 and 12P5/1P1/2P3 and sequenced (n = 6) as previously described [9]. HTLV-1 proviral copy number and beta-globin gene copy number were quantified using real-time quantitative PCR monitored by SYBR Green I dye incorporation in a Roche LightCycler 1.5, using the Tax sequence-specific primers SK43 and SK44. HTLV-1 copy number was estimated by interpolation from standard curves and expressed as a percentage of infected PBMCs.

## Results

Over a 3-year period, in an outpatient setting where over 3000 patients with varying skin conditions are seen, 60 participants, were enrolled. Thirty-three patients were HTLV-1 seropositive, nine of these were co-infected with HIV-1. In addition to the positive clinical findings, 19 patients underwent HTLV DNA PCR and all were confirmed HTLV-1 and therefore fulfilled the study criteria for IDH. Only HTLV-1 infected IDH confirmed subjects are included in the analysis (n = 19) and all HIV-1/HTLV-1 co-infected patients have been excluded from this analysis. The summary of the results is captured in Table 2. The ages at time of testing ranged from 8 months to 15 years. The median age at first presentation/ diagnosis was 8 years (IQR 7–11). The majority of patients (52.9%) were between 6 and 10 years old, 68.4% were female. All were Black African. The scalp (78.9%) and axillae (73.7%) were regions most commonly affected. Only nine patients (47.4%) had chronic nasal discharge or crusting of the anterior nares. Lesional skin cultures were positive for *Staphylococcus aureus* in 55.6% and for Streptococcal *sp* in 33.3%. The streptococcal species were a combination of: *ß-haemolytic streptococcus* (BHS) groups A, B, C and G, together with *Streptococcus pyogenes.* The most common of these was BHS group G. The blood count data revealed anaemia in 20% of the participants. The lymphocyte and platelet counts were within normal ranges, but the ESR was elevated at 40 mm/hr (*normal range* 3-9 mm/hr) in all participants. The immunoglobulin levels that could be measured in our laboratory were raised. The median CD4 count was elevated, with the CD4 count percentage of 56% (*normal range* 36-46% for children) while the CD8 count fell within normal limits, and therefore CD4:CD8 ratio was elevated. The median HTLV-1 proviral load was 10.5% (*range* 1.8-29.8%). All HTLV-1 isolates genotyped were of the Cosmopolitan, Subtype A (HTLV-1a). Of the nine family members tested, five were mothers of the enrolled IDH patients. Four of the 5 mothers had clinical lesions of IDH and were HTLV-1 seropositive. One mother had no features for IDH but HTLV-1 serology was not performed. Initially patients received short courses of antibiotics but this was associated with relapses on stopping treatment. Control was maintained when penicillin-containing oral antibiotics were given simultaneously with mild-to-moderate topical steroids continuously for three to six months.

**Table 2:** IDH study summary data: summary of the analysis of 19 IDH patients

Total enrolled	*n =* 60*		
HTLV-1 +	*n =* 33		
IDH confirmed	*n =* 19		
HTLV-1/HIV +	*n =* 9		
	(of n = 33)		
Ethnicity	all black african		
HTLV-1 subtype	Cosmopolitan (1a)		
Age	mean	8 years	
	range	8 months −15 years (IQR 7–11)	
Gender distribution	male	68.40%	
	female	52.60%	
Microbiology	Staph Aureus	55.60%	
	Streptococcal spp	33.60%	
Clinical examination		dermatological	scalp (78.9%) and axilla (73.7%) commonest sites of involvement
		neurological	no evidence for HAM/TSP
		opthalmological	corneal opacities 3(15.6%)
Immunologic parameters		mean (normals) g/l	
	IgA	3.52 (0.65-2.9)	
	IgG	22.6 (5.2-15.6)	
	IgM	1.38 (0.28-2.4)	
SPEP		mean (normals) g/l	
	Albumin	32.5 (32–50)	
	Alpha 1 globulin	4.0 (1.72-3.30)	
	Alpha 2 globulin	11.1 (4.2-8.7)	
	Beta globulin	11.4 (5.2-10.5)	
	Gamma globulin	26.7 (7.1-14.5)	
Blood counts		mean (normals)	
	Hb	12.3 (11.5-13.5) g/dl	
	WBCC	10.1 (4-11x10^9)	
	Platelets	405 (150-450x10^9)	
	ESR	40 (3–9 mm/hr)	
	CD4 count	1958 (500–1500) cells/mm3	
	CD8 count	1150 (436–2278) cells/mm3	
	CD4 : CD8 ratio	1.7:1	
HTLV-1 proviral load		mean (range%)	
		10.5 (1.8-29.8)	

*remaining patients (n = 27) not further analysed, majority of which had HIV related seborrhoiec dermatitis.

## Discussion

HTLV-1 is endemic in KZN with a reported seroprevalence of 2.6% in the Ngwelezana [10] and of 3.35 in Ubombo (Tarin, 1997) both districts within the same province as Durban. The mean age of our cohort (8 years) was much older than reported in the Caribbean where IDH usually presents between the ages of 2 and 4 years. Perhaps the subtropical climate of South Africa could explain these differences or the disease may be less severe in the early stages resulting in late presentation. The clinical features of IDH in South Africa are similar to that described elsewhere. However, chronic nasal discharge and/or nasal crusting was much less common that expected, (a mandatory criteria for diagnosis). These findings support the suggestion by de Oliveira [11] and Suite et al. [12] who have contested the inclusion of nasal crusting as a major criterion for the diagnosis of IDH. Farre et al., in their evalution of 42 cases from Brazil, records the fact that not all but 30 of their 42 cases, from Brazil exhibited crusting of the nares [13], raising the same issue that crusting around the nostrils should not be mandatory in cases of IDH. There appears to be a major difference between the disease in South Africa and that in Senegal, Brazil and Jamaica.

**Figure 1:**
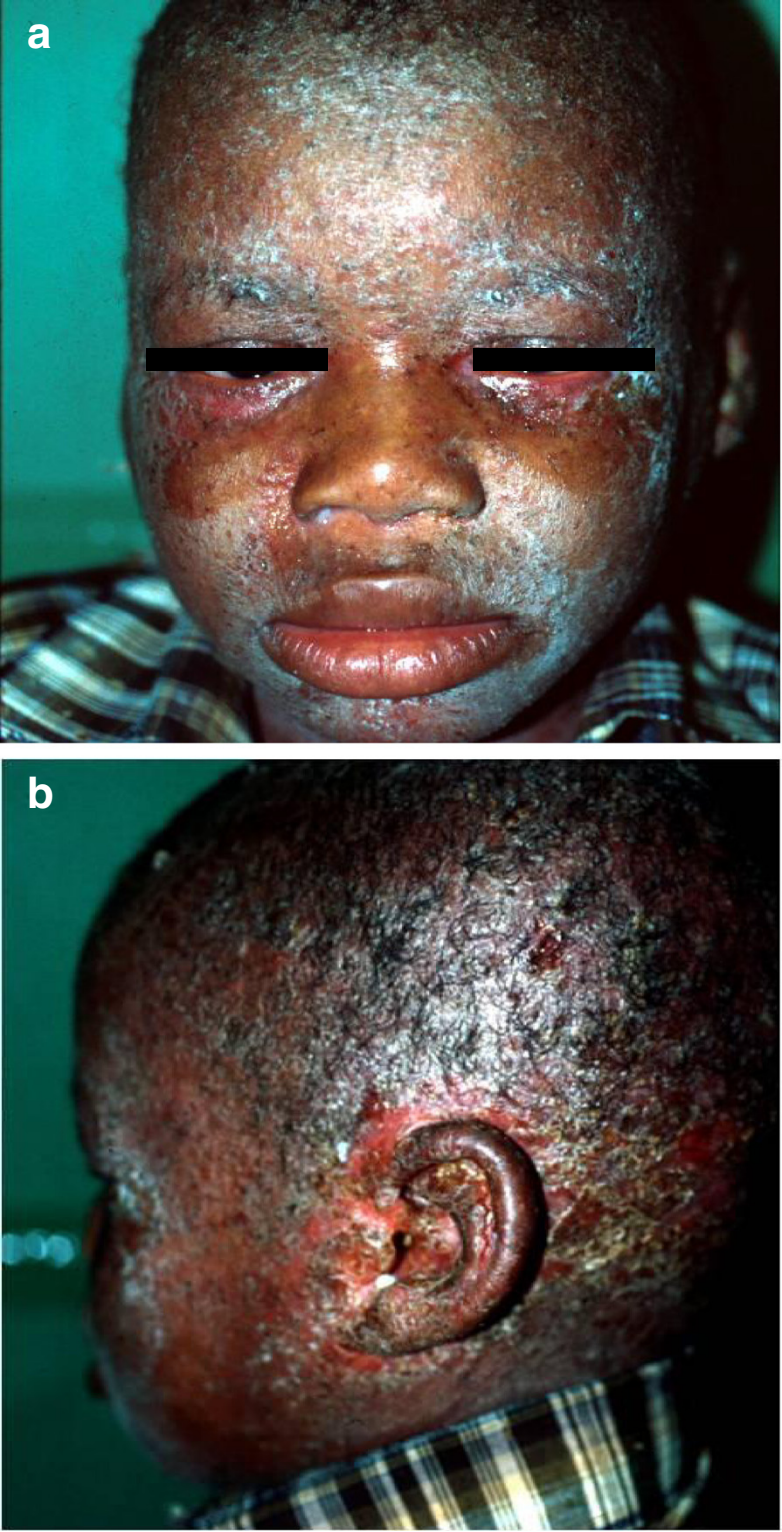
**A typical patient with IDH showing exudative dermatitis with crusting on the face, scalp, external ear, and retro-auricular areas. a**. also demonstrates crusted lesions on face together with blepharitis that characterises IDH in most patients. **b**. Shows an exudative dermatitis with crusting on the face, scalp, external ear, and retro-auricular areas.

**Figure 2:**
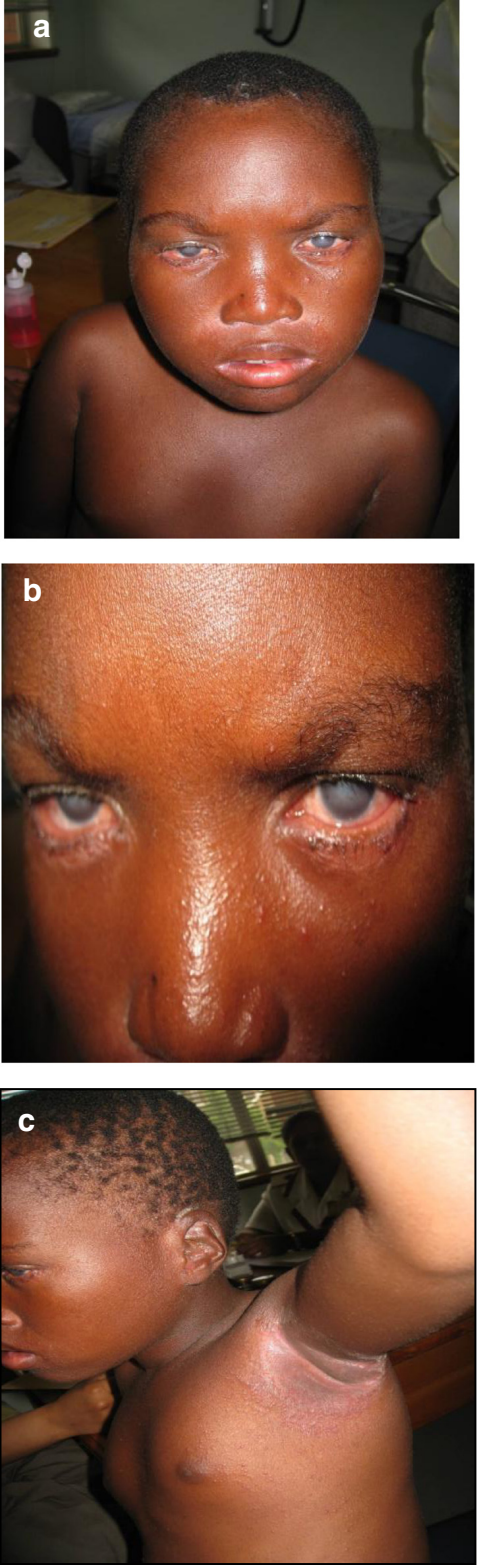
**Another patient with infective dermatitis, presenting with only some of the clinical features of IDH. a**: Infective dermatitis patient with corneal opacities **(b)** that affect a proportion of patients with this condition, also demonstrating flexural involvement **(c)**.

Our cases had less severe IDH presentation than in the those from Senegal where lesions were more infective and crusted whereas the morphology of lesions in our study was more inflammatory than infective, (Figures 1 and 2). Complications have been reported in 30-35% of all patients with IDH [5,14]. However, the cases in our series had a low complication rate (15.6%), none of the cases had other HTLV-1-related complications, such as HAM/TSP nor ATLL. The inconsistency in the presence of a generalized fine papular rash of the original description of IDH, taken together with our findings (the variation in presence of nasal crusting) appear to affirm Mahe and co-worker’s speculation that there are minor variants of IDH which do not show all the features of full-blown IDH as reported in the literature. Clusters of IDH were observed in 4 of the 9 families in this study, reinforcing the hypothesis that HTLV-1 infection, in all but rare cases of IDH, is from mother to child. All six viruses were of the widely prevalent *Cosmopolitan subtype A* (HTLV-1 a), consistent with published data from KZN [15]. We had to exclude nine patients with clinical manifestations of IDH who were co-infected with HIV. Interestingly these were all adults whereas IDH is considered a childhood disease. However, there have been case reports of IDH beginning in adult life [16,17]. The lack of a published scoring system to assess severity of lesions in IDH makes comparison of the clinical appearances of lesions in those co-infected with HIV-1, difficult. It was therefore difficult to determine whether the co-infected cases were truly IDH, or HIV-related seborrhoeic dermatitis.

Except for the absence of anemia in our study population in contrast to the previous reports, other laboratory parameters were in keeping with what has been documented in the previous descriptions.

The data show that cases of IDH had a significantly high HTLV-1 proviral load (mean levels of 10.5%). It has been suggested that IDH act as a cofactor for the development of HTLV-1- associated diseases, such as HAM/TSP and ATLL but the mechanisms are still unclear. HTLV-1 proviral load levels is regarded as important predictor of ATLL and HAM/TSP. It would be interesting to follow all the identified cases with IDH over time and monitor the clinical evolution of the disease to determine if they develop any of the HTLV-1 associated conditions (ATLL, HAM/TSP).

## Conclusions

IDH is a distinct entity that affects the African population of KwaZulu Natal, South Africa. It is predominantly a disease of childhood. The clinical features were in keeping with other series worldwide. Some differences in presentation are noted, particularly later age of onset and less nasal discharge. Interplay between genetic factors and environmental (including socioeconomy and climate) factors, may determine the age of presentation, severity of lesions and therefore explain the variation of IDH presentations between countries. The currect IDH criteria, needs revision. Crusting of the anterior nares should not be mandatory. The revised criteria should take into consideration the heterogeity this disease might have between different geographical locations.

## Competing interests

The authors declare that they have no competing interests.

## Authors’ contributions

CH and AM conceived, designed, realized the study and analysed data. GPT and NG perfomed some of the experiments included in the results of this study. AIB perfomed nuerological examinations. CH, AM and NPK wrote the paper. All authors read and approved the final manuscript.

## Pre-publication history

The pre-publication history for this paper can be accessed here:

http://www.biomedcentral.com/1471-5945/13/11/prepub
